# Association between anti‐dementia medications and long‐term clinical outcomes in Alzheimer's disease dementia: A nationwide population‐based study

**DOI:** 10.1002/alz70860_102460

**Published:** 2025-12-23

**Authors:** Yun Jeong Hong, Si‐Baek Lee, Seong Hoon Kim, Jeong‐Wook Park, Mina Kim

**Affiliations:** ^1^ Catholic University of Korea, Uijeongbu St. Mary's Hospital, Uijeongbu, Korea, Republic of (South); ^2^ the Catholic University of Korea, Uijeongbu, Korea, Republic of (South); ^3^ Uijeongbu St. Mary's Hospital, Uijeongbu, Korea, Republic of (South); ^4^ The Catholic Univ. of Korea Uijeongbu St. Mary's Hospital, Uijeongbu, Korea, Republic of (South); ^5^ Hanmi Pharm.Co., Seoul, Korea, Republic of (South)

## Abstract

**Background:**

Anti‐dementia medications are prescribed in Alzheimer's disease (AD). However, there have been frew studies that clarified long‐term outcomes according to dose and drug compliance. We investigated whether clinical outcomes, represented by incidences of moderate to severe dementia, institutionalization, or death, are different according to doses/ drug compliance in AD patients using big data from the National Health Insurance Service (NHIS).

**Method:**

We examined a population‐based cohort obtained from the NHIS Senior Cohort (NHIS‐SC) data that comprises approximately half a million recipients of medical insurance in South Korea during 2009‐2022. The medication information (acetylcholiesterase inhibitors and memantine) was analyzed with respect to treatment dose and persistence across 12 years. From the initial cohort of 1,704,547 patients diagnosed with dementia between 2010 and 2016, 486,398 patients were included in the analysis. To compare clinical outcomes, patients were divided into two groups according to the following categories: 1) drug compliance during the first 3 years: those with a medication possession ratio (MPR) below 70% vs those with 70% or more, 2) anti‐dementia medication dose during the first 3 years: those with low‐dose medications vs those with optimal dose (standard dose according to clinical guideline), 3) optimal treatment during the first 3 years: those receiving optimal treatments (MPR ≥70% with optimal dose) vs those with sub‐optimal treatments (low‐dose or low (<70%) MPR). All statistical analyses were performed using SAS Enterprise Guide (version 8.3; SAS Institute, Cary, NC, USA).

**Result:**

Optimal drug compliance (MPR≥70%) group (*n* = 66103) showed better long‐term clinical outcomes (fewer death/ institutionalization/ lower progression rates) than those in low drug compliance group (*n* = 64332). Optimal dose group (*n* = 66103) showed better long‐term clinical outcomes than those in low dose group (*n* = 34771). Optimal treatment group (*n* = 66103) showed better long‐term clinical outcomes than those in sub‐optimal treatment group (*n* = 420295). Progression to moderate to severe dementia within 5 years and death were related with baseline age, gender, drug compliance and optimal anti‐dementia medications during the first 3 years from dementia diagnosis.

**Conclusion:**

Long‐term clinical outcomes in AD dementia patients are different according to optimal doses of anti‐dementia medications/ drug compliance during the first 3 years from dementia diagnosis.

